# Cycloastragenol as an Exogenous Enhancer of Chondrogenic Differentiation of Human Adipose-Derived Mesenchymal Stem Cells. A Morphological Study

**DOI:** 10.3390/cells9020347

**Published:** 2020-02-03

**Authors:** Marta Anna Szychlinska, Giovanna Calabrese, Silvia Ravalli, Nunziatina Laura Parrinello, Stefano Forte, Paola Castrogiovanni, Elisabetta Pricoco, Rosa Imbesi, Sergio Castorina, Rosalia Leonardi, Michelino Di Rosa, Giuseppe Musumeci

**Affiliations:** 1 Department of Biomedical and Biotechnological Sciences, Anatomy, Histology and Movement Sciences Section, School of Medicine, University of Catania, 95123 Catania, Italysilviaravalli@gmail.com (S.R.); pacastro@unict.it (P.C.); roimbesi@unict.it (R.I.); mdirosa@unict.it (M.D.R.); 2 Department of Biomedical and Biotechnological Sciences, University of Catania, 95123 Catania, Italy; giovanna.calabrese@unict.it; 3 Division of Hematology, AOU “Policlinico-Vittorio Emanuele”, 95125 Catania, Italy; lauraparrinello@tiscali.it; 4 IOM Ricerca, 95029 Viagrande, Italy; stefano.forte@grupposamed.com; 5 Department of Medical and Surgical Sciences and Advanced Technologies, Anatomic Pathology Section, School of Medicine, University of Catania, 95124 Catania, Italy; elisabettap@tiscali.it; 6 Department of Medical, Surgical and Advanced Technological Sciences “G.F. Ingrassia”, University of Catania, 95124 Catania, Italy; sergio.castorina@unict.it; 7 Department of General Surgery and Medical-Surgical Specialties, University of Catania, 95124 Catania, Italy; rleonard@unict.it; 8 Research Center on Motor Activities (CRAM), University of Catania, 95123 Catania, Italy; 9 Department of Biology, Sbarro Institute for Cancer Research and Molecular Medicine, College of Science and Technology, Temple University, Philadelphia, PA 19122, USA

**Keywords:** cartilage regeneration, tissue engineering, cycloastragenol, human adipose-derived mesenchymal stem cells, chondrocyte phenotype, hypertrophy

## Abstract

Stem cell therapy and tissue engineering represent a promising approach for cartilage regeneration. However, they present limits in terms of mechanical properties and premature de-differentiation of engineered cartilage. Cycloastragenol (CAG), a triterpenoid saponin compound and a hydrolysis product of the main ingredient in Astragalus membranaceous, has been explored for cartilage regeneration. The aim of this study was to investigate CAG’s ability to promote cell proliferation, maintain cells in their stable active phenotype, and support the production of cartilaginous extracellular matrix (ECM) in human adipose-derived mesenchymal stem cells (hAMSCs) in up to 28 days of three-dimensional (3D) chondrogenic culture. The hAMSC pellets were cultured in chondrogenic medium (CM) and in CM supplemented with CAG (CAG–CM) for 7, 14, 21, and 28 days. At each time-point, the pellets were harvested for histological (hematoxylin and eosin (H&E)), histochemical (Alcian-Blue) and immunohistochemical analysis (Type I, II, and X collagen, aggrecan, SOX9, lubricin). After excluding CAG’s cytotoxicity (MTT Assay), improved cell condensation, higher glycosaminoglycans (sGAG) content, and increased cell proliferation have been detected in CAG–CM pellets until 28 days of culture. Overall, CAG improved the chondrogenic differentiation of hAMSCs, maintaining stable the active chondrocyte phenotype in up to 28 days of 3D in vitro chondrogenic culture. It is proposed that CAG might have a beneficial impact on cartilage regeneration approaches.

## 1. Introduction

Articular cartilage shows poor regenerative properties, due to its alymphatic, avascular, aneural nature and the limited cellularity of the tissue, composed of chondrocytes sparsely embedded within the collagen and proteoglycan-based extracellular matrix (ECM) [1]. Changes in biomechanical and metabolic features of articular cartilage, related to aging or injury, lead to matrix degradation, resulting in progressive tissue degeneration and causing severe pain and disability of the joint. An inadequate tissue repair, followed by a progressive loss of cartilage and remodeling of the underlying subchondral bone, leads to osteoarthritis (OA), a very common form of severe degenerative articular cartilage disease [2].

OA is a multifactorial disease, but aging represents the most prominent risk factor contributing to its development. It has been shown that one of the most important age-related epigenetic factors associated with OA is accelerated telomere shortening [3]. The latter implies that OA chondrocytes lose their ability to synthesize matrix components and begin to synthesize proteins, which contribute to ECM degradation [4].

Current cartilage repair treatment approaches help to repair the articular cartilage lesions and reduce pain in affected joints, but only to some degree and are considered ineffective [5,6]. The limitations of cartilage repair itself, coupled with inadequate clinical strategies and rising incidence rates of OA, have compelled cell-based therapies for sustained recovery of the functional properties of native tissue [7].

Mesenchymal stem cells (MSCs) have emerged as a promising cell source for cartilage repair. Major advantages of using MSCs for cartilage regeneration are due to their easy availability, proliferative capacity, and multilineage potency [7,8]. However, the current cartilage engineering techniques still present limitations for clinical application. The major challenges are represented by phenotypic instability after implantation, poor integration into cartilage defects and the surrounding tissues, and the insufficient mechanical properties of the engineered cartilage that do not replicate the properties of the native tissue. Accumulating data from literature demonstrated that during long-term culture, MSCs undergo spontaneous transformation, showing typical features of senescence, including the premature and progressive telomere shortening, which results in cell loss and impaired regenerative potential [9]. These features resemble a similar pattern activated in chondrocytes, leading to OA development, as stated above. Moreover, inflammation in the osteoarthritic joints is another barrier to overcome for successful cartilage repair. MSC behavior can be influenced by multiple exogenous microenvironmental stimuli that act as chondrogenic inducers [10,11]. As an external signal, the mechanical forces represent an important modulator of the native articular cartilage metabolic activities and serve to maintain the cartilage homeostasis [12]. Bioactive molecules are other chondrogenesis-enhancing factors. Among these, transforming growth factor-βs (TGF-βs), insulin-like growth factor-I (IGF-I), fibroblast growth factor (FGF) and bone morphogenetic proteins (BMPs) are reported to stimulate the proteoglycan synthesis, improve the repair of osteochondral defects, and enhance the MSC-based chondrogenesis. In addition, cell–cell and cell–ECM interactions and adhesion properties are crucial for maintaining cell phenotype and for inducing effective MSC-chondrogenesis [1]. 3D cell culture methods facilitate these interactions, allowing cells to create an “in vivo-like” microenvironment and better preserve the stem cell phenotype and to reproduce the native tissue morphology [13,14]. Native hyaline cartilage development starts with the migration and condensation of mesenchymal progenitor cells differentiating into active chondrocytes [15]. Cartilage formation from self-assembling MSCs in vitro recapitulates important cellular events during mesenchymal condensation that precedes native cartilage development. In pellet culture, cells form spherical aggregates that deposit matrix and grow over time [16,17,18,19].

Cycloastragenol (CAG) is a triterpenoid saponin compound and a hydrolysis product of astragaloside IV, the main active ingredient of Astragalus membranaceus Bunge (Leguminosae). It has been used for centuries as an important medicine to reinforce vital energy, strengthen superficial resistance and promote the growth of new tissues. An increasing body of evidence indicates that CAG has a wide spectrum of pharmacological functions such as wound healing, and anti-apoptotic and anti-tumor activities, which are attracting attention in the research community [20,21,22]. Among the most interesting pharmacological effects of CAG, that catch attention as a potential chondrogenic enhancer and anti-osteoarthritic molecule, are represented by telomerase activation, telomere elongation, and anti-inflammatory and anti-oxidative properties [23].

In addition, CAG has been proven to possess anti-arthritic properties and to suppress joint inflammation via the inhibition of IL 1β, TNF α, and iNOS production in the arthritis rat model [24,25,26,27].

For these reasons, the present study aimed to investigate, for the first time in literature, the effects of CAG on chondrogenic differentiation of human adipose-derived MSCs (hAMSCs) cultured as self-assembling 3D spherical aggregates, in terms of maintenance of stable chondrocyte phenotype, anti-hypertrophic property, and ECM component deposition.

## 2. Materials and Methods

### 2.1. Cycloastragenol

Cycloastragenol (CAG) (purity ≥ 98%) was purchased from Sigma-Aldrich (St. Louis, MO, USA). The CAG powder has been dissolved in dimethyl sulfoxide (DMSO) to make stock solutions and then it has been stored at −20 °C.

### 2.2. Cell Culture and Phenotypic Characterization

Three human visceral adipose tissue biopsies of 10–15 g of weight, harvested from three different donors (two women aged 52 and 56, and one man aged 54), have been used in the present study to isolate hAMSCs, as previously reported [28].

The samples have been supplied by the Mediterranean Institute of Oncology (IOM) (Viagrande, Italy) under an approved Institutional Review Board protocol (project ID code: 829_1 of 8 February 2013, IOM Institutional Review Board). The hAMSCs were characterized by flow cytometry and immunofluorescence analyses using positive (CD105 and CD90), and negative (CD45 and CD34) mesenchymal stem cells surface markers. For flow cytometry, cells (80% of confluence) were detached with 0.05% trypsin/EDTA and washed in PBS 1X. 1 × 10^4^ cells/tube were stained with the following antibodies: CD105 PE (clone1G2), CD90 FITC (clone F15.42.1.5), CD45 FITC (clone J.33), CD34 PE (clone 581), and respective isotopic controls according to manufacturer indications. All antibodies were purchased from Beckman Coulter (Milano, Italy). All tubes were incubated in the dark for 20 min at room temperature. Cells were then washed with PBS 1X and finally analyzed by flow cytometry using an FC-500 five-color flow cytometer (Beckman Coulter, Pasadena, CA, USA). For each tube, 1000 events were acquired. CXP Analysis software (Beckman Coulter˝, Inc.) was used for data analysis. Immunofluorescence analysis was assessed on hAMSCs seeded in eight-well BD Falcon culture slides at the density of 5 × 10^3^ cells/cm^2^. hAMSCs were fixed in 4% paraformaldehyde (PFA), permeabilized in 0.4% Triton and then blocked by incubation in 5% goat serum for 1 h. The primary incubation was assessed with the following anti-human antibodies: mouse CD105 (1:50, Novus Biologicals, Littleton, CO, USA), mouse CD90 (1:50, Santa Cruz Biotechnology, Dallas, TX, USA), rabbit CD45 (1:100, Epitomics, Burlingame, CA, USA), and rabbit CD34 (1:100, Epitomics) overnight at 4°C. The following day slides were washed and incubated with the appropriate secondary AlexaFluor antibodies (1:2000, LifeTechnologies Italia, Monza, Italy) for 2 h at room temperature. Nuclei were counterstained with DAPI (1:5000) for 5 min. Finally, slides were mounted in fluorescent mounting medium Permafluor (Thermo Scientific, Waltham, MA, USA) and images were acquired using a Leica DMI4000B fluorescence microscope (Leica, Wetzlar, Germany).

### 2.3. CAG Cytotoxicity Assay

CAG cytotoxicity for hAMSCs was assessed by the MTT assay (Roche Life Science), which measures the conversion of 3-[4,5-dimethylthiazol-2-yl]-2,5 diphenyl tetrazolium bromide to formazan crystals by living cells, which process determines the mitochondrial activity that can be photometrically detected (492 nm). The hAMSCs were seeded at 1 × 10^4^ cells/cm^2^ in flat-bottomed 96-well plates, in 100 μL of basal culture medium (DMEM, low-glucose, Carlo Erba, Milan, Italy) with 2 mM L-glutamine, 1% pen/strep and 10% Fetal Bovine Serum. After 24 h, cells were cultured in different conditions: (1) basal medium and (2) basal medium supplemented with different concentrations of CAG (0.1/0.1/1/10/25 μM). To obtain CAG solutions at micromolar concentrations the CAG–DMSO stock solution has been highly diluted in basal medium (1:800–1:2000). The metabolic activity was then assessed by MTT conversion for 24 and 48 h. A blank control comprising the only medium was also included. For each measurement, three replicates per condition were included.

### 2.4. Three-Dimensional Pellet Cultures of hAMSCs

2.5 × 10^5^ hAMSCs/tube/mL were seeded in 2.5  mL polypropylene tubes. Cell pellets were formed by centrifugation (390× *g* for 5 min) and cultured in basal medium (BM)—Dulbecco’s Modified Eagle’s Medium (DMEM, low-glucose, Carlo Erba, Milan, Italy) with 2 mM L-glutamine, 1% pen/strep and 10% Fetal Bovine Serum (BSA), in a CO_2_ incubator at 37 °C. Tube lids were loosely maintained on top of each tube to allow gas exchange. After 24 h, the pellets were cultured in different conditions in triplicate: (1) chondrogenic medium (CM), MSC chondrogenic differentiation medium, (PromoCell, Heidelberg, Germany); (2) CM supplemented with CAG (0.1 μM), for 7, 14, 21, and 28 days. The media were changed twice per week during 28 days of culture.

### 2.5. Analysis of Pellet Size

The size of the pellets was analyzed at 7, 14, 21, and 28 days. The cellular pellets presented an ellipsoidal shape and their surface at different time points was calculated as follows: the histological sections (objective lens: 2.5×) of the middle part of pellets, stained by hematoxylin and eosin (H&E) have been used. The pellet surface has been calculated by using an image-analysis software (AxioVision Release 4.8.2-SP2 Software, Carl Zeiss Microscopy GmbH, Jena, Germany) and expressed in μm^2^. Image analysis was performed on four pellets per condition.

### 2.6. Histological Analysis

At 7, 14, 21, and 28 days, cell pellets were harvested and histologically processed as previously described [29]. The pellets were rinsed in PBS, fixed in 10% buffered formalin (Bio-Optica, Milan, Italy). After an overnight wash, specimens were dehydrated in graded ethanol, cleared in xylene, and paraffin-embedded. After wax infiltration, pellets were orientated in the cassettes in the same direction. Sections (5 μm thick) were cut from paraffin blocks using a rotary manual microtome (Leica RM2235, Milan, Italy), mounted on silane-coated slides (Menzel-Gläser, Braunschweig, Germany) and stored at room temperature. Afterwards, the sections were dewaxed in xylene, hydrated by graded ethanol, and stained by H&E to evaluate cell morphology and the presence or absence of morphological alterations. The slides were examined with a Zeiss Axioplan light microscope (Carl Zeiss, Oberkochen, Germany), and pictures were taken with a digital camera (AxioCam MRc5, Carl Zeiss, Oberkochen, Germany).

### 2.7. Analysis of sGAGs by Histochemistry

The sections were obtained as previously described. Alcian Blue (Bio-Optica, Milan, Italy) was used to assess synthesis of sulphated glycosaminoglycans (sGAGs) containing proteoglycans in cell pellets. The assessment was made by computerized densitometric measurements as reported below. The sections were examined with a Zeiss Axioplan light microscope (Carl Zeiss, Oberkochen, Germany) and photomicrographs were captured using a digital camera (AxioCam MRc5, Carl Zeiss, Oberkochen, Germany).

### 2.8. Immunohistochemistry

For immunohistochemical analysis, cell pellets were processed as previously described [30]. Briefly, the slides were dewaxed in xylene, hydrated using graded ethanols, and incubated for 30 min in 0.3% H_2_O_2_/PBS to quench endogenous peroxidase activity before being rinsed for 20 min with PBS (Bio-Optica, Milan, Italy). The sections were heated (5 min × 3) in capped polypropylene slide-holders with citrate buffer—pH 6 (pH 6.0; Bio-Optica, Milan, Italy) or Tris-EDTA buffer (pH 8.0; Bio-Optica, Milan, Italy), using a microwave oven (750 W, LG Electronics Italia S.p.A., Milan, Italy) to unmask antigenic sites. Following antigenic retrieval, the sections were incubated overnight at 4 °C with diluted rabbit polyclonal antibodies against type I collagen (ab34710; Abcam, Cambridge, UK), type II collagen (ab34712; Abcam, Cambridge, UK), and type X collagen (ab58632; Abcam, Cambridge, UK), rabbit monoclonal anti-SOX9 (ab185966; Abcam, Cambridge, UK), rabbit polyclonal anti-lubricin antibody (ab28484; Abcam, Cambridge, UK), and anti-aggrecan (ab3778; Abcam, Cambridge, UK) diluted 1:100 in PBS (Sigma-Aldrich, Milan, Italy). Immune complexes were then treated with biotinylated link antibodies (horseradish peroxidase polymer (HRP)-conjugated anti-rabbit and anti-mouse were used as secondary antibodies) and then detected with peroxidase-labelled streptavidin, both incubated for 10 min at room temperature (LSAB + System-HRP, K0690, Dako, Glostrup, Denmark). Immunoreactivity was visualized by incubating the sections for 2 min in 0.1% 3,3′-diaminobenzidine (DAB) (DAB substrate Chromogen System; Dako, Glostrup, Denmark). The sections were lightly counterstained with Mayer’s hematoxylin (Histolab Products AB, Göteborg, Sweden), mounted in Glycerol Vinyl Alcohol (GVA) (Zymed Laboratories, San Francisco, CA, USA), observed with an Axioplan Zeiss light microscope (Carl Zeiss, Oberkochen, Germany), and photographed with a digital camera (AxioCam MRc5, Carl Zeiss, Oberkochen, Germany).

### 2.9. Computerised Densitometric Measurements and Image Analysis

Four pellets per group, the area of each of which was about 3,500,000 Pixel^2^, were analyzed for histochemical evaluation of Alcian Blue staining, which detects mucosubstance content (GAGs) and to quantify the level of immunostaining of positive anti-Col I, anti-Col II, anti-Col X, anti-aggrecan, anti-SOX9, and anti-lubricin antibodies immunolabeling. It was used as an image-analysis software (AxioVision Release 4.8.2-SP2 Software, Carl Zeiss Microscopy GmbH, Jena, Germany), which quantifies the level of staining in the densitometric count (pixel^2^) normalized to the area of each section expressed in pixel^2^. Digital micrographs were taken using the Zeiss Axioplan light microscope (Carl Zeiss, Oberkochen, Germany), using a lens with a magnification of ×10, i.e., total magnification 100) fitted with a digital camera (AxioCam MRc5, Carl Zeiss, Oberkochen, Germany). Three blinded investigators (two anatomical morphologists and one histologist) made the evaluations that were assumed to be correct if the recorded values had no statistically significant difference. If disputes concerning interpretation occurred, a unanimous agreement was reached after sample re-evaluation.

### 2.10. Statistical Analysis

The statistical analysis was performed using GraphPad Instat^®^ Biostatistics version 3.0 software (GraphPad Software, Inc., La Jolla, CA, USA). Datasets were tested for normal distribution with the Kolmogorov–Smirnov test. All variables were normally distributed. Ordinary one-way-ANOVA (Tukey’s multiple comparisons test) was used for comparisons between more than two groups. The *p*-values <0.05 were considered statistically significant; *p*-values >0.05 were considered not significant (ns). The data are presented as the mean ± SD.

## 3. Results

### 3.1. CAG Cytotoxicity in hAMSCs

**Figure 1:**
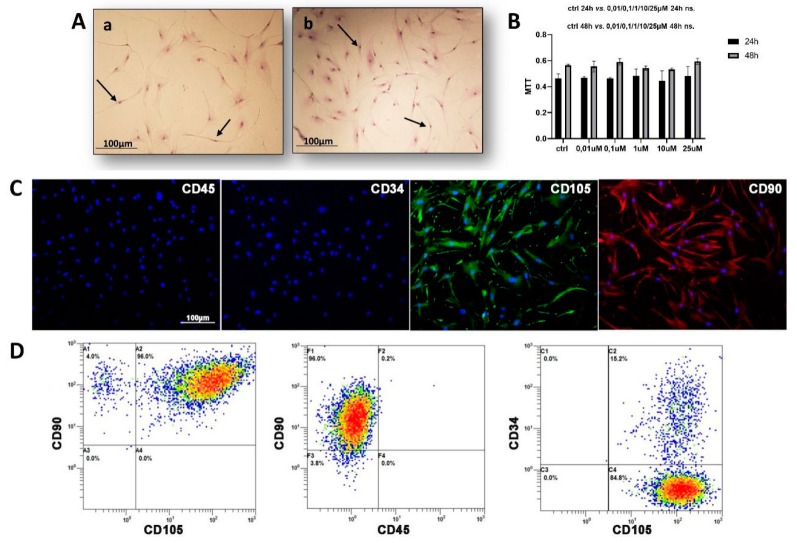
(**A**) The micrographs (a,b) of adipose tissue-derived mesenchymal stem cells (AMSCs) cultured in basal medium at different passages (Aa: p. 10; Ab: p. 12), evidenced by May–Grünwald Giemsa staining. The AMSCs show a characteristic fibroblast-like morphology (black arrows). Objective lens: 2.5×, scale bar: 100 μm. (**B**) Cycloastragenol (CAG) cytotoxicity for AMSCs cultured with basal medium (CTRL) and with different concentrations of CAG (0.01/0.1/1/10/25 μM), assessed by the MTT assay at 24 h and 48 h. See the Materials and Methods section for details. Results were presented as the mean ± SD. ANOVA was used to evaluate the significance of the results. (**C**) Phenotypic characterization of human AMSCs (hAMSCs) by immunofluorescence and (**D**) flow cytometry of negative hematopoietic markers (CD45 and CD34) and positive MSC surface markers (CD105 and CD90). In C) CD105 expression is stained in green and CD90 in red. The hAMSC nuclei are counterstained with DAPI (blue). Power magnification: 20×. Scale bar: 100 μm.

The cytotoxicity of CAG in hAMSCs was addressed. The mitochondrial activity was evaluated through MTT assay during a 48 h period after CAG addition and compared with control cultures. The results show that CAG was not cytotoxic for hAMSCs at any of the selected concentrations (Figure 1B). CAG concentration was maintained at 0.1 μM to conduct the following study.

### 3.2. hAMSC Characterization

The images of hAMSCs at passages 10 (Figure 1Aa) and 12 (Figure 1Ab) cultured in BM and evidenced by May–Grünwald Giemsa staining, were analyzed. The cells showed a characteristic fibroblast-like morphology (Figure 1A), which represents their normal aspect in two-dimensional (2D) conditions.

After isolation, immunofluorescence and flow cytometry analyses have been performed on hAMSCs using two negative (CD45 and CD34) and two positive (CD105 and CD90) MSC surface markers. The results of both analyses have confirmed that hAMSCs were negative for CD34 and CD45, whereas they expressed high levels of CD105 and CD90 (Figure 1C,D).

### 3.3. Histological Analysis of hAMSC 3D Cell Pellets

To promote the production of cartilaginous ECM, the hAMSCs were cultured in three-dimensional (3D) conditions. After centrifugation, the hAMSCs were able to form cell aggregates (pellets) within 24 h. Subsequently, the pellets were switched and cultured in CM that served as a control and in CAG-supplemented CM (as described in the Materials and Methods section) during 28 days of culture.

**Figure 2:**
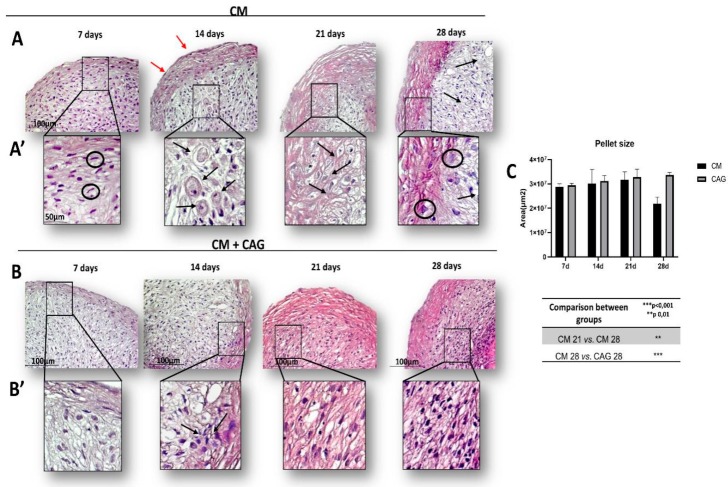
Time-based chondrogenic differentiation of hAMSC pellets, either without (**A**) or with (**B**) the supplementation of CAG (0.1 µM), evidenced by hematoxylin and eosin (H&E) staining at 7, 14, 21 and 28 days. (**A**) control group (chondrogenic medium: CM); (**B**) CAG-supplemented group (CM + CAG). (**A’**,**B’**) The inserts representing the image magnifications (objective lens, 20×; scale bar: 50 μm), to evidence the morphology changes observed in a time-dependent manner. In the control group (**A’**) we observe: at 7 days, fibroblast-like cell shape (black circles), high cell ability to aggregate and form 3D pellets, not much evident extracellular matrix (ECM); at 14 days, some cells show the chondrocyte-like rounded morphology and presence of lacunae (black arrows), and external perichondral capsule formation (red arrows); at 21 days the pellets exhibit the cartilage-like morphology: the cells are organized into columns, lacunae are well-evidenced (black arrows) and ECM production increases; at 28 days the pellets show decreased ECM deposition, especially in the middle zone (black arrows), an evident thickening of the external capsule and cell enlargement (black circles), indicative of hypertrophy. In the CAG-supplemented group (**B’**), the pellets at 7 and 14 days show a similar morphology when compared to the seven-days control pellets (**A’**), seemingly with the increased cell proliferation activity (black arrows); at 21 days, the pellets show an increased cell proliferation and ECM deposition activity, evidenced by the intense H&E staining; at 28 days, the cells within the pellets seem to maintain their phenotypic stability, suggested by the enhanced ECM deposition, apparent increase of cell proliferation activity, and poor cellular hypertrophy phenomena. The increased cell proliferation, cellular aggregations, and ECM deposition activity, exhibiting a cartilage tissue-like morphology are observed in the CAG-supplemented group (**B’**). Objective lens, 10×; scale bar: 100 μm. (**C**) The pellet-size analysis was assessed by image-analysis software and expressed in μm^2^. See the Materials and Methods section for details. Results were presented as the mean ± SD. ANOVA was used to evaluate the significance of the results. ** *p* < 0.01; *** *p* < 0.001.

The H&E staining was used to observe the morphology of 3D hAMSCs after chondrogenic differentiation at different time points: 7, 14, 21, and 28 days, in the presence of CM (control, Figure 2A) and CAG-supplemented CM (Figure 2B). Our data show that the cells within the control pellets, after seven days of chondrogenic differentiation, are able to form aggregates and 3D structure, even if their internal organization is still disordered (Figure 2A). After 14 days, some cells within the pellets show the chondrocyte-like rounded morphology and the deposition of fibers parallel to the pellet surface (Figure 2A). At 21 days the cells are organized into columns that represent a continuum of chondrocyte differentiation and they are located into lacunae (Figure 2A). At 28 days of CM culture, the pellets show an involution activity represented by cell enlargement, indicative of hypertrophy and evident thickening of the external capsule (Figure 2A). In CAG-supplemented CM conditions (Figure 2B), the pellets at 7 and 14 days show a similar morphology relative to the seven-days control pellets (Figure 2B). At 21 days, the pellets show the cartilage-like morphology (Figure 2B). At 28 days of CAG–CM culture, the cells within the pellets seem to maintain their phenotypic stability, suggested by poor cellular hypertrophy phenomena (Figure 2B). Overall, CAG shows to increase cellular aggregation, exhibiting a cartilage tissue-like morphology up to 28 days of 3D culture. These results have been confirmed by the pellet size analysis (Figure 2C).

### 3.4. Analysis of sGAG Production by hAMSC 3D Pellet Cultures

**Figure 3:**
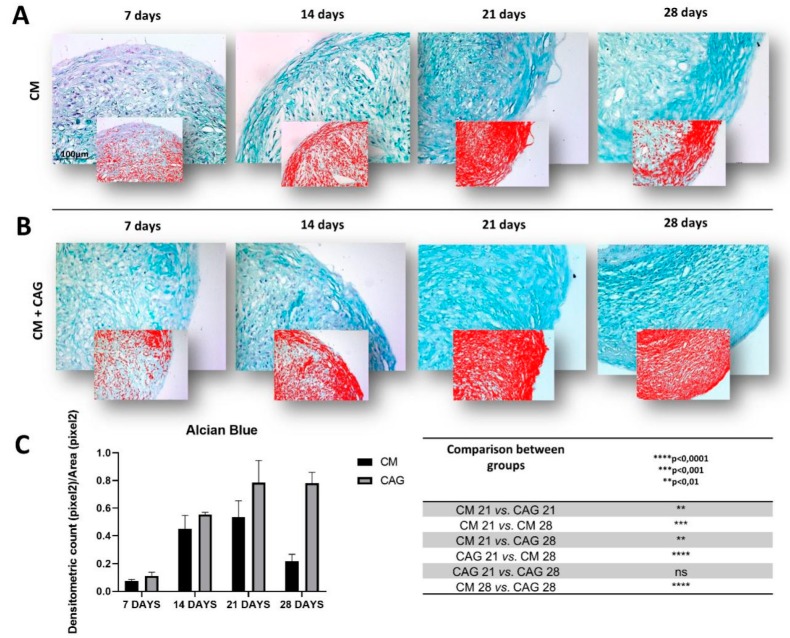
Histochemical evaluation of the mucosubstance content glycosaminoglycan (GAGs) in 3D AMSC-derived chondropellets at 7, 14, 21, and 28 days of culture by Alcian Blue staining through computerized densitometric measurements and image analysis. (**A**) control group (chondrogenic medium: CM); (**B**) CAG-supplemented group (CM + CAG). The smaller attached images represent the image analyses by the software: red color corresponds to high-intensity Alcian Blue staining confirming the sGAG synthesis. (**C**) Graph representing the level of staining expressed as densitometric count (pixel^2^) normalized to the area of each section expressed in pixel^2^. Results were presented as the mean ± SD. ANOVA was used to evaluate the significance of the results. ** *p* < 0.01; *** *p* < 0.001; **** *p* < 0.0001; ns, not significant. See the Materials and Methods section for details. (**A**,**B**) Objective lens, 10×; scale bar: 100 μm.

To evaluate the effects of CAG on the production of cartilaginous matrix deposition and sGAG content, the pellet sections were analyzed through Alcian Blue staining after 7, 14, 21, and 28 days of CM culture with and without CAG supplementation. The sGAG deposition is represented as intense blue within pellet ECM. Experimental data obtained using image-analysis software, which quantifies the level of Alcian Blue staining expressed as densitometric count (pixel^2^)/area(pixel^2^) report that the control pellets cultured in CM show a linear increase in sGAG deposition up to 21 days of culture (Figure 3A). At 28 days, a significant decrease (*p* < 0.001) in sGAG and ECM deposition is observed within the control pellets when compared to 21-day control pellets (Figure 3C), confirming the histological analysis (Figure 2A). On the contrary, the pellets cultured with the CAG-supplemented CM, show a linear increase in sGAG deposition up to 28 days of culture (Figure 3B) and a significant increase (*p* < 0.0001) in sGSG deposition when confronted with 28-day control pellets (Figure 3C), confirming the histological analysis (Figure 3B). Moreover, the statistical analysis, when compared to the controls, demonstrate that CAG significantly enhances (*p* < 0.01) the sGAG deposition within the ECM at 21 days of culture (Figure 3C).

### 3.5. Analysis of SOX9, Col II, Col I, Col X, Aggrecan, and Lubricin Expression in Chondrogenic hAMSC 3D Pellet Cultures

**Figure 4:**
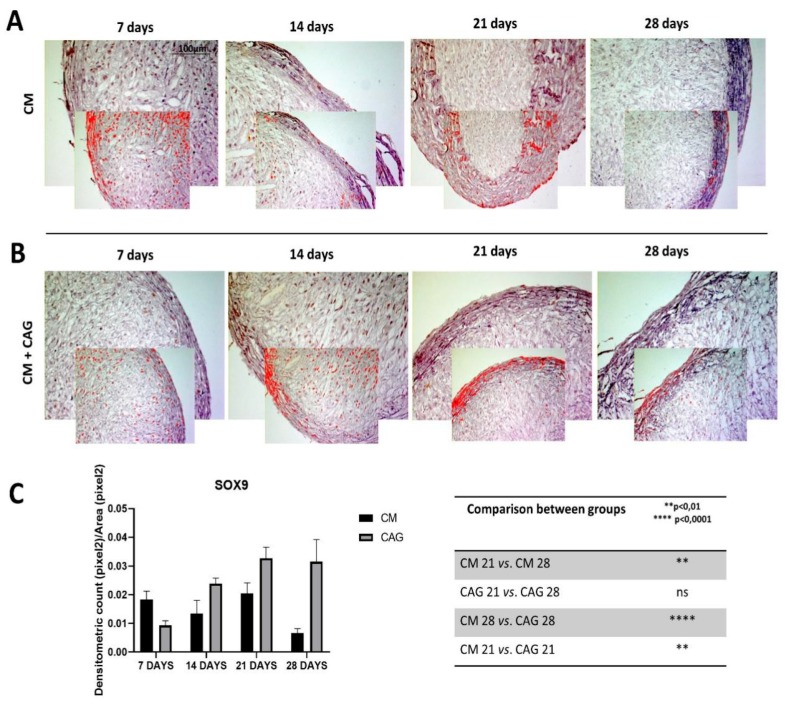
SOX9 immunohistochemical evaluation in 3D AMSC-derived chondropellets at 7, 14, 21, and 28 days of culture through computerized densitometric measurements and image analysis. (**A**) control group (chondrogenic medium: CM); (**B**) CAG-supplemented group (CM + CAG). The smaller attached images represent the image analyses by the software: red color corresponds to brown staining (immune complexes labelled with chromogen). (**C**) Graph representing the level of staining expressed as densitometric count (pixel^2^) normalized to the area of each section expressed in pixel^2^. Results were presented as the mean ± SD. ANOVA was used to evaluate the significance of the results. ** *p* < 0.01; **** *p* < 0.0001; ns, not significant. See the Materials and Methods section for details. (**A**,**B**) Objective lens, 10×; scale bar: 100 μm.

**Figure 5:**
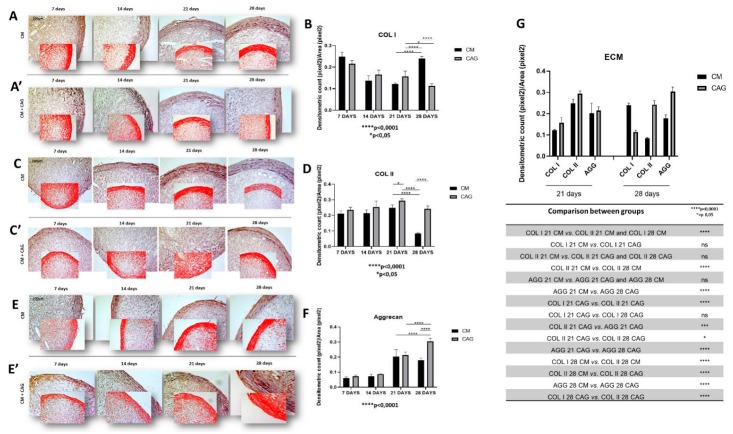
(**A**) Col type I, (**C**) Col type II, and (**E**) aggrecan immunohistochemical evaluation in 3D AMSC-derived chondropellets at 7, 14, 21, and 28 days of culture through computerized densitometric measurements and image analysis. (**A**,**C**,**E**) Control groups (chondrogenic medium: CM); (**A’**,**C’**,**E’**) CAG-supplemented groups (CM + CAG). The smaller attached images represent the image analyses by the software: red color corresponds to brown staining (immune complexes labelled with chromogen). (**B**,**D**,**F**) Graphs representing the level of respective immunolabelings expressed as densitometric count (pixel^2^) normalized to the area of each section expressed in pixel^2^. Objective lens, 10×; scale bar: 100 μm. (**G**) Graph representing the comparison of densitometric count (pixel^2^) of ECM components (Col I, Col II, AGG) immunolabeling identified among groups and compared between them at 21 and 28 days of 3D culture. All results were presented as the mean ± SD. ANOVA was used to evaluate the significance of the results. * *p* < 0.05; *** *p* < 0.001, **** *p* < 0.0001; ns, not significant. See the Materials and Methods section for details.

In the present study, the expression of SOX9 is significantly higher in CAG-supplemented pellets, respectively at 21 (*p* < 0.005) and 28 (*p* < 0.0001) days of culture (Figure 4A–C), when compared to the controls. Col type I was expressed in early condensed mesenchymal bodies mainly observed at 7 and 14 days and, subsequently, reduced and expressed especially at the perichondral margin (external capsule) at 21 days of 3D culture both in CM-cultured pellets and CAG-supplemented ones (Figure 5A). At 28 days of culture, there is a significant difference in the expression of Col I when compared to 21-day pellets, which appeared further reduced (*p* < 0.05) in CAG-supplemented pellets (Figure 5A,B) and highly increased (*p* < 0.0001) in the control ones (Figure 5A,B). This latter is also probably due to the thickening of the perichondral margin in the control pellets where we can see the accumulation of Col type I deposition in the superficial zone and some degenerative phenomena in the inner zone.

Seemingly, Col type II is highly expressed in the hAMSC-derived pellets up to 21 days of culture both in CM (Figure 5C) and CAG-supplemented CM conditions (Figure 5C), suggesting the cartilage formation. Contrarily to the Col I expression, at 28 days of 3D culture the Col II expression decreases drastically (*p* < 0.0001) in CM conditions (Figure 5C,D), while in CAG-supplemented 28 day pellets it decreases less significantly (*p* < 0.05) and it remains comparable to the 21-day pellets of the same group (Figure 5C,D). The expression of aggrecan follows the similar expression profile of Col type II, even if in lesser amounts (Figure 5E,F).

The expression profiles of the ECM cartilage structural molecules, represented by Col type I, Col type II and aggrecan, are compared in Figure 5G, taking into account two most representative time-points (21 and 28 days), in which the most significant changes between the control and the experimental pellets are seen. Due to the 3D sphere culture, all the factors dissolved into the medium probably need to diffuse within the pellets to reach the inner zone, which might explain the differences in chondrogenic markers detection between the inner and external zones of the pellets. The ECM components expression follows the expression profile of SOX9 (Figure 4A–C).

**Figure 6:**
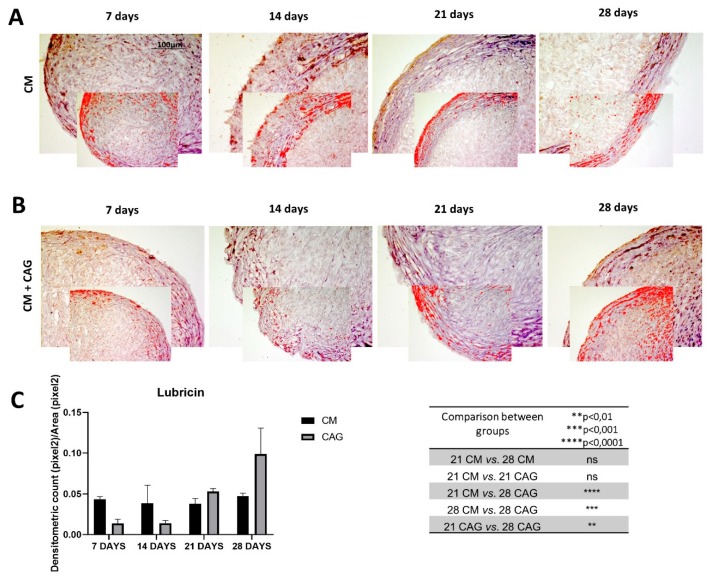
Lubricin immunohistochemical evaluation in 3D AMSC-derived chondropellets at 7, 14, 21, and 28 days of culture through computerized densitometric measurements and image analysis. (**A**) control group (chondrogenic medium: CM); (**B**) CAG-supplemented group (CM + CAG). The smaller attached images represent the image analyses by the software: red color corresponds to brown staining (immune complexes labelled with chromogen). (**C**) Graph representing the level of staining expressed as densitometric count (pixel^2^) normalized to the area of each section expressed in pixel^2^. Results were presented as the mean ± SD. ANOVA was used to evaluate the significance of the results. ** *p* < 0.01; *** *p* < 0.001; **** *p* < 0.0001; ns, not significant. See the Materials and Methods section for details. Objective lens, 10×; scale bar: 100 μm.

Furthermore, the expression of lubricin within the pellets has been evaluated. The results demonstrate the increase in lubricin expression in a time-dependent manner, especially in the superficial pellet zone, up to 28 days of 3D culture, both in CM and CAG-supplemented conditions (Figure 6A–C). At 28 days, lubricin expression profile is similar to that of SOX9, Col II and aggrecan. When compared to the 21-day control pellets, it increases in a not-significant manner in the CM pellets, while it increases significantly (*p* < 0.05) in the CAG-supplemented pellets, both in the superficial and inner pellet zone. Therefore, we can notice a very significant increase (*p* < 0.001) of its expression when compared to 28-day control pellets (Figure 6C), confirming the data reported above.

**Figure 7:**
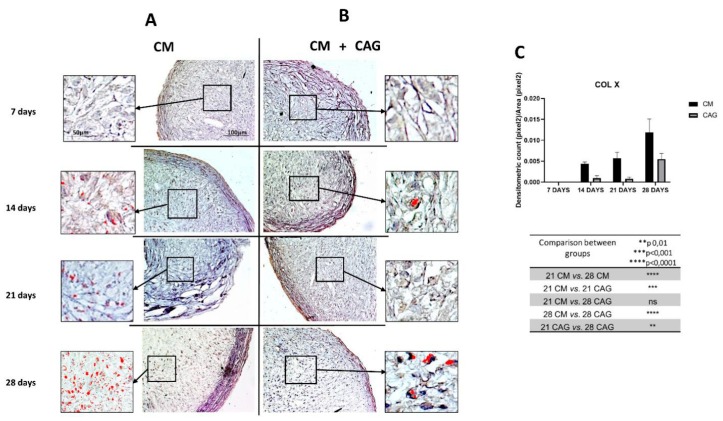
Col type X immunohistochemical evaluation in 3D AMSC-derived chondropellets at 7, 14, 21, and 28 days of culture through computerized densitometric measurements and image analysis. (**A**) control group (chondrogenic medium: CM); (**B**) CAG-supplemented group (CM + CAG). The inserts represent the image magnifications (objective lens, 20×; scale bar: 50 μm), to evidence the image analyses by the software in the pellet middle zone: red color corresponds to brown staining (immune complexes labelled with chromogen). (**C**) Graph representing the level of staining expressed as densitometric count (pixel^2^) normalized to the area of each section expressed in pixel^2^. Results were presented as the mean ± SD. ANOVA was used to evaluate the significance of the results. ** *p* < 0.01; *** *p* < 0.001; **** *p* < 0.0001; ns, not significant. See the Materials and Methods section for details. Objective lens, 10×; scale bar: 100 μm.

Finally, the expression of Col type X (Figure 7A–C) has been assessed to evaluate the premature cell entry into hypertrophy under the in vitro cell culture conditions. The results demonstrated that pellets cultured in CM undergo hypertrophic de-differentiation at 28 days of the in vitro culture (Figure 7A), highlighted by the increased Col X expression (*p* < 0.0001) when compared to the 21-day control (Figure 7A,C). On the contrary, the pellets supplemented with CAG at 28 days of culture (Figure 7B) demonstrate a significantly lower (*p* < 0.0001) expression profile of Col type X when compared to the 28-day control pellets (Figure 7C).

## 4. Discussion

It is well-known that, from the embryological point of view, the cartilage is the first skeletal tissue to be formed and it participates in endochondral ossification within skeletal development. It is characterized by chondrogenesis and subsequent bone formation, which is a multi-developmental process regulated by dynamic morphogenetic and phenotypic changes [31]. It has been well-established that, at certain point of chondrogenic differentiation in vitro, the cells enter into hypertrophy, losing their active chondrocyte phenotype and becoming less suitable for cartilage regeneration approaches, representing the main challenge for their clinical application [32]. Tissue engineering offers possibilities for optimization of cartilage regeneration by combining MSCs, 3D cell culture conditions, mechanical stimuli, growth factors, and biomolecules for the support of neocartilage formation [11]. Although several procedures have been developed, no standardized protocol has yet been established and in vitro cartilage regeneration presents several limits. Therefore, the application of the existing knowledge regarding the multifactorial aspects of OA disease, complex articular cartilage structure, chondrogenesis enhancing factors, and exogenous stimuli, aimed at developing phenotypically stable engineered cartilage is a promising outcome of the future research.

CAG is a bioactive *Radix Astragali*-derived triterpene aglycone, which has been demonstrated to have various pharmacological actions including regenerative and wound healing effects [20,33], anti-aging effects through telomerase activation [21], anti-fibrosis [34], anti-apoptosis [35], and anti-inflammation [22]. According to existing in vitro and in vivo studies, CAG is safe, and it did not induce any toxic or genotoxic effects, as confirmed by the result of bacterial reverse mutation assay, in vitro chromosome aberration assay, and in vivo erythrocyte micronucleus assay [36].

In the present study, the ability of CAG to enhance hAMSC chondrogenesis and maintain the phenotypic stability of the neocartilage up to 28 days of culture has been investigated. The results of the histological analysis show a good cell condensation and aggregation capacity in both CM and CAG-supplemented groups, which formed spherical pellets already at seven days of 3D culture and maintained this condition up to 28 days. In parallel, the same experiment on MSCs cultured in basal medium (DMEM without chondrogenic factors) in the same experimental conditions has been conducted, but several difficulties in the inclusion of the obtained micro-masses has been encountered, since the cells did not undergo aggregation process and no pellets have formed. For this reason, it was impossible to conduct parallel morphological analysis on these samples, which have been excluded from the study.

In CM conditions, at seven days the pellets resemble the prechondrogenic mesenchyme tissue, evidenced by high expression of Col type I, confirming data from developmental studies reporting that it is highly expressed in early chondrogenesis [37,38,39,40,41,42]. The latter has then been further confirmed by the SOX9 expression [37], in both CM and CAG-supplemented pellets (Figure 4A–C). Data from literature indicate that SOX9 expression starts in the prechondrogenic mesenchyme and is sustained at high levels in fully differentiated chondrocytes where it directs Col type II and aggrecan deposition [43]. At 14 days of CM culture, a differentiation into chondrocytes and fibril structure formation within pellets were more evident in the CM group, while the CAG-supplemented group showed the morphological features of prechondrogenic mesenchyme, suggesting that CAG might delay the chondrogenesis developmental stages in vitro. These last results were also confirmed by the SOX9 expression profile. At 21 days of chondrogenic culture, a deposition of cartilaginous ECM and cartilage formation were shown in both groups (Figure 5G). However, the ECM synthesis and cell proliferation were more evident in the CAG-supplemented group, underlined by the H&E and Alcian Blue strong stainings and high ECM component expression (Figure 2B,C, Figure 3B,C and Figure 5G). Col type I to Col type II transition can be seen at this time point, suggesting chondrogenic differentiation and confirming data from the literature [44]. The interesting results come from the SOX9 expression profiles, where the increased Col type II and aggrecan deposition is not accompanied by the sustained upregulation of SOX9. Similar results have been observed in a study by Murdoch et al., which stated that it appears probable that the expression of this gene in the prechondrogenic mesenchyme stage is already sufficient to support the ECM component deposition in later stages of differentiation [44]. Seemingly, it is conceivable that there are other mechanisms that complement or enhance the activity of SOX9 transcription during chondrogenic differentiation. This aspect should be certainly further studied. From the morphological point of view, at this time point, we can notice a reasonably uniform matrix deposition within the pellets in both groups. However, in some pellets, we observed the formation of the external fibrous capsule at the perichondral margin, with the main deposition of collagen fibers, especially in the CM control pellets, and the increased ECM deposition within the internal pellet zone. This might be due to the dissimilar culture medium-associated factor diffusion distances due to the spherical shape of the pellets. The latter may also determine the differences in the cell density and oxygen concentration within the pellets. At 28 days of culture, we can notice the most important and evident differences between the experimental and control groups. The hypertrophy and degradation of cartilage ECM are marked in the CM group, evidenced by the histological and immunohistochemical analysis. On the contrary, at the same time point, the CAG-supplemented group demonstrated the morphologic features similar to the 21-day pellets, poor hypertrophic de-differentiation process, and absence of degenerative phenomena (Figure 2B). At this time point, we can also notice an overexpression of lubricin (Figure 6B), an important chondrocyte marker [39]. These results confirm the data from literature, which report that the overexpression of SOX9 in human MSCs leads to enhanced proliferative, biosynthetic, and chondrogenic activities, and a reduction or delay in the hypertrophic differentiation of the cells [42]. Overall, according to these results, we can state that the CAG-supplemented group, especially at 21 and 28 days of 3D chondrogenically induced culture, showed an enhanced metabolic activity, emphasized by the enhanced cartilaginous ECM deposition, increased lubricin expression, and delayed chondrogenic de-differentiation with the maintenance of the phenotypically stable chondrocyte up to 28 days of 3D culture. Certainly, these preliminary results suggest that there would be value in pursuing this study and investigating the CAG’s effects on hMSCs for longer periods and from different sources.

## 5. Conclusions

Clinical application of engineered cartilage faces challenges, derived from phenotypic instability of the cells in long-term cultures, and poor neo-tissue morphological and regenerative properties. A comprehensive understanding of signaling molecules involved in chondrogenesis and their actions on MSC-derived neocartilage, as well as the studies of exogenous enhancers, able to overcome the stated challenges, became crucial. The results of the present study demonstrate that CAG improves a chondrogenic differentiation of hAMSCs, maintaining stable the active chondrocyte phenotype up to 28 days of 3D in vitro culture. Therefore, it is proposed that CAG might have a beneficial impact on future approaches for cartilage regeneration.
